# Recent developments of mesenchymal stem cell-derived extracellular vesicles in respiratory system diseases: A review

**DOI:** 10.1097/MD.0000000000043416

**Published:** 2025-07-18

**Authors:** Tingting Zu, Meng Gao, Baohe Liu, Xuejing Zhang, Fuling Wu

**Affiliations:** a Pediatrics Department, Binzhou Medical University Hospital, Binzhou 256600, China.

**Keywords:** extracellular vesicles, mesenchymal stem cells, respiratory system diseases, review

## Abstract

The emergence of extracellular vesicles with nanostructure characteristics signifies a novel acellular therapeutic approach at the nanoscale, influencing cellular processes such as proliferation, differentiation, and apoptosis, as well as serving as vehicles for precise drug delivery. Substantial evidence supports their involvement in tissue regeneration, immune modulation, targeted tumor diagnosis and therapy effects. This review seeks to establish a theoretical framework for investigating the impact of extracellular vesicles derived from mesenchymal stem cells on respiratory system disorders in future research.

## 1. Introduction

The latest statistics published by the World Health Organization indicate that by 2048, noncommunicable diseases will account for over 90% of all deaths, with respiratory diseases representing a significant concern. This highlights a substantial deficiency in the effective management and treatment of respiratory diseases on a global scale, where targeted therapy and precise drug delivery systems are of paramount importance. In comparison to oral and intravenous administration, the latest research indicates that nano-carrier drug delivery systems exhibit enhanced bioavailability and more robust targeting capabilities, which have garnered the attention of researchers.^[1,2]^

Stem cells (SCs) have the potential to serve as effective carriers or targeted delivery systems for nanomedicine, enabling the directed delivery and release of drugs through nanotechnology. A multicenter phase 2a randomized trial conducted in the United States has demonstrated the high safety and significant efficacy of intravenous injection of SCs for patients suffering from acute respiratory distress syndrome (ARDS).^[3]^ Moreover, research has demonstrated that targeted therapy using mesenchymal SCs facilitates lung function recovery and delays or inhibits the progression of ARDS to multiple organ injury.^[4]^ Significant advancements have been made in the utilization of stem cell therapy for respiratory diseases, with promising prospects for future applications.^[5]^ Nevertheless, the low engraftment and survival rates of mesenchymal SCs in the lungs have constrained their therapeutic potential.^[4]^

## 2. Mesenchymal stem cell-derived extracellular vesicles

Further research has demonstrated that the therapeutic effects of mesenchymal SCs are contingent upon their capacity to secrete EVs. Based on the release pathway, size, content, and function, they can be classified into 3 main subtypes: extracellular vesicles (EVs), apoptotic bodies, and microvesicles.^[6]^ These EVs can transfer a range of molecules and organelles, including mRNA, microRNA, proteins, lipids, and even mitochondria, to target cells and tissues. This enables them to alter the gene expression of target cells, alleviate inflammatory reactions, and exert their therapeutic effects.

Mesenchymal stem cell-derived EVs are nano-sized EVs, with a diameter typically ranging from 50 to 200 nm. EVs offer several advantages over mesenchymal SCs, including low immunogenicity, non-tumorigenicity, high stability, the capacity to cross the blood-brain barrier, and ease of storage.^[7–9]^ Extracellular vesicles possess a multitude of biological functions, which are driven by various mechanisms^[10]^ and involved in regulating processes such as cell proliferation, differentiation, and apoptosis. Additionally, they have been demonstrated to facilitate vascular regeneration and tissue repair, regulate the immune system, and exhibit antitumor activity. Extracellular vesicles may also be employed as a targeted drug carrier in clinical treatment, representing a novel research focus in the domain of non-cell therapy and offering a vast scope for the advancement of innovative therapeutic and intervention strategies. Recent animal studies and clinical trials have substantiated the safety and efficacy of EVs.^[4,11]^

The selection of EVs isolation technology constitutes a pivotal factor in the quality of EVs preparation, as it determines the final yield and biological activity of EVs, thereby directly impacting the clinical therapeutic effect. In order to ensure the stability and clinical reproducibility of EVs preparations, it is particularly important to establish strict quality control standards, the core indicators of which include the purity and specificity of EVs and the removal efficiency of non-vesicular impurities. However, a paucity of uniform standard operating procedures for EVs isolation and purification persists on a global scale, thereby impeding the clinical translation of EVs therapy to a certain extent. This study offers a systematic comparison of the advantages and limitations of current mainstream separation techniques (Table 1).^[12–16]^ In accordance with the Minimal Information for Studies of EVs guidelines established by the International Society for EVs, comprehensive characterization of EVs serves as a cornerstone for evaluating isolation methodologies. This process not only quantifies the purity and yield of isolated EVs but also rigorously confirms their identity. Specifically, the characterization of extracellular vesicles encompasses 3 primary dimensions: physical properties, molecular marker, and functional assessment (Table 2).^[17,18]^

**Table 1:** Systematic evaluation of mainstream EVs isolation technologies.

Isolation methods	Advantages	Disadvantages
Differential ultracentrifugation	Simple operation; wide range of applications	Cell line-dependent; low purity; prone to sample damage.
Density gradient ultracentrifugation	Relatively high purity; EVs integrity preservation; effective in removing residual non-vesicular contaminants from differential ultracentrifugation prepurified EV samples	The isolation of extracellular vesicles is frequently achieved through density gradient centrifugation, which utilizes sucrose or iodixanol to differentiate vesicles. However, this method is accompanied by the potential for prolongation of the processing time and the risk of compromised sample yield due to material retention in gradient layers
Immunomagnetic bead capture method	High purity; specific isolation	Low production; expensive
Tangential flow filtration	High purity; lateral flow is applied during filtration to prevent particle accumulation and pore clogging	Expensive; complex equipment
Size exclusion chromatography	Relatively high purity; EVs integrity preservation	Column capacity constraints; contamination susceptibility
Aptamer-based microfluidic biosensors	Seamless integration; portability; Enables high-sensitivity detection of specific exosome subpopulations in ultra-low sample volumes (1 aL–1 nL), while minimizing reagent and sample consumption	The development of high-affinity aptamers targeting extracellular vesicles surface proteins remains nascent, and exposure to bioactive reagents may destabilize sensor interfaces

EVs = extracellular vesicles.

**Table 2:** Essential characterization of EVs.

Analytical dimension		Core metrics	Standardized methods and technical specifications
Physical properties		1. Size distribution (50–200 nm)	1. NTA
		2. Morphological structure (cup-shaped bilayer)	2. TEM
		3. Particle-to-protein ratio	3. TRPS
Molecular marker	Positive identification markers	1. Tetraspanins	1. WB
		2. ESCRT-associated proteins	2. FC
		3. Tissue-specific markers	3. MS
	Negative exclusion markers	1. Organelle contaminants: calnexin, cytochrome C, histone H3	Negative control assay
		2. Apoptotic bodies	
Functional assessment		1. Cellular uptake efficiency	1. Fluorescent tracking assay 2. In vitro functional models
		2. Targeting delivery capacity	
		3. Functional bioactivity assessment	

FC = flow cytometry, MS = mass spectrometry, NTA = nanoparticle tracking analysis, TEM = transmission electron microscopy, TRPS = tunable resistive pulse sensing, WB = Western blotting.

## 3. The application of extracellular vesicles in respiratory system diseases

**Figure 1.**
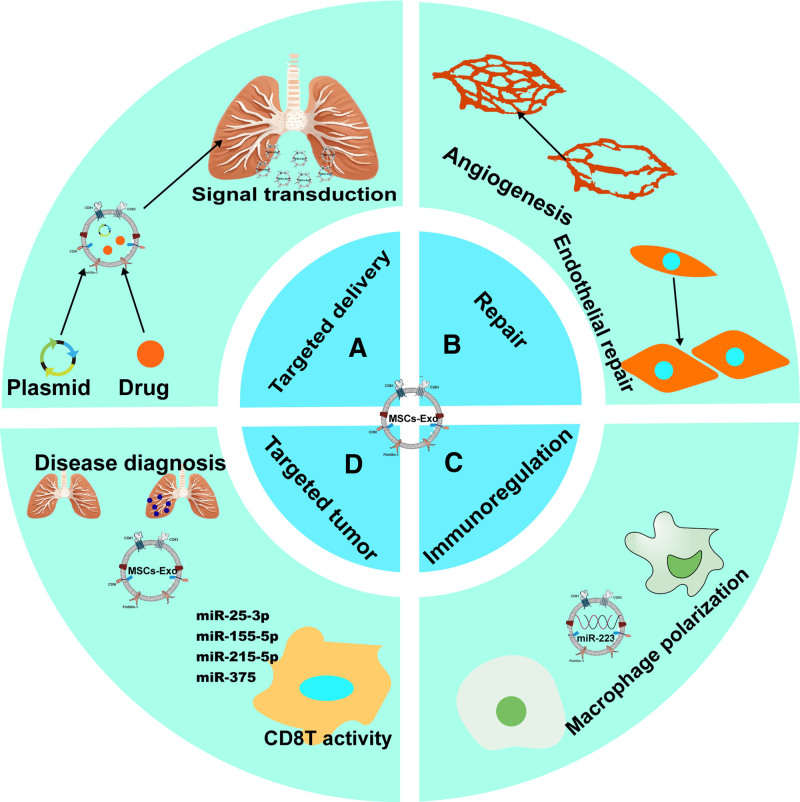
Multiple functions of EVs: (A) EVs play a role in intercellular signaling, and those secreted by mesenchymal SCs have the capacity to carry plasmids and drugs to target cells, thereby exerting a regulatory effect. (B) EVs promote vascular regeneration and regulate fibroblasts to promote tissue repair. (C) EVs can orchestrate immune responses, particularly by inducing macrophage polarization, which plays a pivotal role in both inflammatory regulation and tissue homeostasis. (D) EVs have emerged as valuable liquid biopsy biomarkers for tumor detection. Their cargo, especially microRNAs, exhibits significant therapeutic potential by regulating T-cell proliferation and exerting direct antitumor effects. EVs = extracellular vesicles, SCs = stem cells.

At present, mesenchymal stem cell-derived EVs have been employed in a range of disease models.^[19]^ A substantial body of evidence substantiates their notable therapeutic efficacy in addressing diverse pulmonary lesions, thereby establishing them as a highly promising cell-free alternative therapeutic modality (Fig. 1).^[20]^ For example, in a lipopolysaccharide-induced acute lung injury cell model,^[21,22]^ studies have demonstrated that EVs can reverse lung damage by downregulating the nuclear factor erythroid 2-related factor 2 and antioxidant response element factors. Furthermore, they can markedly enhance the NF-κB signaling pathway, facilitating its interaction with nuclear factor κB kinase subunit β and preventing the progression of acute lung injury into a more severe and complex condition like ARDS.

Furthermore, EVs derived from lung cancer exhibit a distinctive expression profile of microRNAs and messenger RNAs, which enhances the transcriptional activity of TCF4/β-catenin and activates the Wnt signaling pathway.^[23]^ Furthermore, they facilitate immune evasion, epithelial-mesenchymal transition, and angiogenesis,^[24]^ thereby playing a crucial role in the formation and metastasis of lung cancer. Exosome therapy has been demonstrated to effectively inhibit bleomycin-induced lung fibrosis progression and significantly reduce the expression of β-catenin and cellular senescence markers in mouse models.^[25]^ Moreover, additional research has demonstrated that EVs derived from bronchial epithelial cells can inhibit the TGF-β-WNT signaling pathway, thereby suppressing the development of lung fibrosis. Abbaszadeh et al^[26]^ provided a comprehensive overview of the pivotal role of EVs in chronic obstructive pulmonary disease (COPD) and asthma models. They posited that EVs can downregulate inflammatory factors, reduce cell apoptosis, and facilitate lung function restoration and alveolar remodeling. Emerging evidence in the field of bronchopulmonary dysplasia (BPD) research indicate that human umbilical cord mesenchymal stem cell-derived Exosomes (hUC-MSC-Exos) may improve alveolarization deficits by regulating autophagy in alveolar type II epithelial cells.^[27,28]^ Mechanistically, hUC-MSC-Exos activate the Wnt5a/ROCK1 signaling axis, which enhances lamellar body biogenesis and pulmonary surfactant synthesis. These processes are critical for maintaining alveolar structural integrity and respiratory function. This targeted modulation of AT2 cell homeostasis rectifies aberrant alveolar development and establishes a robust preclinical rationale for employing hUC-MSC-Exos as a cell-free regenerative strategy in neonatal lung injury, particularly for mitigating BPD progression in preterm infants.

### 3.1. Targeted drug delivery carrier

Nano-sized EVs possess specific homing targets and can encapsulate various therapeutic drugs, enabling direct interaction with the intracellular environment. Moreover, EVs demonstrate the capacity to undergo membrane remodeling, thereby facilitating precise targeting and transfer of drugs. Research has demonstrated their considerable potential as nano-carriers in targeted antiviral drug therapy, rendering them an optimal candidate for targeted drug delivery.^[8,20]^ The combination of a nanoparticle delivery system with gene therapy methods, such as the use of EVs as carriers to deliver CRISPR-Cas9 plasmids into target cells, may facilitate gene editing and further enhance targeted noninvasive treatment strategies.^[29]^ For example, in a study on the 2019 novel coronavirus, EVs were demonstrated to function as nano-carriers for antiviral therapy.^[7]^

The therapeutic efficacy of extracellular vesicle-based targeted delivery systems is crucially determined by 3 interdependent parameters: the selection of drug-loading strategies, the preservation of EV membrane integrity during cargo incorporation, and the controlled release kinetics of therapeutic payloads. To facilitate the selection of rational technologies for clinical translation, this study provides a comparative analysis of the different EVs drug-carrying technologies (Table 3).^[30,31]^ The scientific optimization of the drug delivery strategy of MSC-EVs is paramount to enhancing clinical efficacy. To that end, systematic studies are required to compare the efficacy of different routes of administration (including oral administration, transdermal administration, intravenous injection, nebulized inhalation, and intraperitoneal injection).^[32]^ Furthermore, the optimization of the delivery modalities has been demonstrated to be a pivotal factor influencing the effectiveness of the treatment (Table 4).

**Table 3:** The functional mechanisms and characteristics of different EV-mediated drug delivery technologies.

Drug delivery technologies	Mechanisms	Characteristics
Genetically engineered endogenous modification	EVs deliver gene-editing tools to target cells, enabling direct genetic engineering modifications designed for parental cells	High specificity and low off-target rate, but costly
Exosome-liposome hybrid vesicles	EVs fuse with liposomes to form hybrid vesicles	Liposomes exhibit relatively high cytotoxicity
Chemical conjugation of drugs to exosome membrane surfaces	Chemical modification via covalent conjugation: Functional groups are added to EVs surfaces using reactive reagents	Risk of compromised membrane structural integrity
Exosome incubation method	Co-incubation of extracellular vesicles with small molecules allows passive diffusion-based drug-loading into EVs over a defined timeframe	Low loading efficiency, often requiring combination with other methods
Electroporation method	Electric field application temporarily disrupts exosome membranes, enhancing transmembrane drug transport	Higher drug-loading efficiency, but risks damaging exosome membrane structure and causing content leakage

EVs = extracellular vesicles.

**Table 4:** The comparison of EVs delivery modalities.

Delivery modalities	Absorption mechanism	Advantages	Disadvantages	Applicable diseases
Oral administration	Gastrointestinal absorption	User-friendly operation with high patient compliance	Gastric acid degradation and intestinal enzymatic digestion	Inflammatory bowel diseases, metabolic diseases
Transdermal administration	Follicular/sweat gland permeation	Localized high concentration	Stratum corneum barrier limitation	Skin injuries
Intravenous injection	Systemic circulation distribution	Rapid onset with system-wide delivery	Nonspecific distribution	Cancers, systemic inflammatory disorders, cardiovascular diseases
Nebulized inhalation	Alveolar-capillary direct absorption	High pulmonary accumulation	Requires prevention of airway deposition	COPD, pulmonary fibrosis, COVID-19
Intraperitoneal injection	Transperitoneal absorption	Localized high concentration, ideal for peritoneal diseases	Invasive procedure with potential risk of peritoneal adhesion	Hepatocellular carcinoma, peritoneal metastatic cancers, peritonitis

EVs = extracellular vesicles.

### 3.2. Promotion of vascular regeneration and tissue repair

Vascular regeneration: it has been demonstrated that EVs are capable of producing hepatocyte growth factor, a matrix metalloproteinase inducer, and vascular endothelial growth factor. These factors have been shown to stabilize the vascular endothelial barrier and promote vascular regeneration.^[7,11]^

The process of tissue repair: an excess of protease activity has been demonstrated to result in lung injury and damage. Alpha-1 antitrypsin (AAT) has the capacity to diminish protease activity, thereby preventing lung tissue damage resulting from protease imbalance and reducing the likelihood of bacterial infections. This, in turn, serves to suppress inflammatory responses. Proteomic studies have demonstrated that the secretome of adipose-derived mesenchymal SCs, comprising soluble factors and EVs, contains AAT, in addition to 72 proteins implicated in protease/antiprotease equilibrium and 46 proteins involved in bacterial responses. Furthermore, experiments have demonstrated that alterations in serum starvation and chemical stimulation (such as IL-1β) can enhance the expression of the AAT-encoding gene, indicating that under specific circumstances, greater AAT production may be achievable. This offers valuable insights into the potential use of EVs in the management of lung disorders associated with AAT deficiency.^[33]^ Moreover, research has shown that EVs can enhance the clearance of sodium-dependent alveolar fluid,^[34]^ thereby facilitating the resolution of lung edema and reducing lung injury. In an in vitro model of hypoxia-induced lung epithelial injury,^[20]^ treatment with conditioned media from mesenchymal SCs was observed to improve cell viability, reduce hypoxic cell injury, and promote tissue repair and cellular homeostasis. Furthermore, EVs have been observed to carry Ang-1 mRNA, which can be transferred to injured pulmonary endothelial cells, thereby promoting endothelial repair.^[9]^ Additionally, they are capable of secreting keratinocyte growth factor, which facilitates the repair of lung epithelial cells.^[7]^

### 3.3. Immunomodulation

EVs have been demonstrated to possess anti-inflammatory and immune-regulatory properties, exerting influence over both innate and adaptive immune system cells. They have been observed to inhibit the proliferation of NK cells, B cells, and T cells, as well as suppress the differentiation and migration of dendritic cells.^[20]^ It has been demonstrated that the immune-regulatory properties of EVs are not determined by genetics but rather induced by inflammatory microenvironments. For example, pro-inflammatory stimuli have been shown to enhance the immune suppression function of EVs derived from adipose-derived mesenchymal SCs.^[35]^ EVs achieve their immune-regulatory function through many different mechanisms, including the transfer and presentation of antigen peptides, DNA-induced cGAS-STING signaling, manipulation of exosomal microRNA gene expression, and signaling induction by exosomal surface ligands.^[10]^ Extracellular vesicles have been demonstrated to play a role in immune regulation by regulating cell-mediated immunity and cytokine release. For instance, they have been shown to reduce the proliferation and apoptosis of CD4 + T cells while increasing the ratio of regulatory T cells to effector T cells.^[8]^

EVs have been demonstrated to contribute to immune regulation by modulating the release of antimicrobial factors and upregulating antimicrobial action mediated by monocytes/macrophages. The antimicrobial activity of these cells is due in part to immune-regulatory substances secreted by EVs that activate and enhance the immune system’s antimicrobial activity. Additionally, exosome-secreting peptides have been observed to exhibit direct antimicrobial activity,^[20]^ which has the potential to enhance the clearance of bacteria, thereby reducing the inflammatory reactions caused by pathogens and subsequent tissue damage.^[34]^ Additionally, they can enhance the secretion of anti-inflammatory cytokines in peripheral blood monocytes. This is exemplified by the upregulation of inhibitory cytokines IL-10 and TGF-β1 in monocytes of asthmatic patients,^[11]^ which in turn promotes Treg proliferation and enhances immune suppression ability. Moreover, EVs can downregulate the expression of pro-inflammatory factors, including prostaglandin E2 and programmed death ligand-1 (PD-L1), thereby inhibiting the activation and infiltration of CD4 T and Th17 cells.^[11]^ This process contributes to the regulation of the immune microenvironment.^[9]^ Additionally, they can serve as a novel cell therapy for the treatment of patients with coronavirus disease 2019 (COVID-19), reversing the cytokine storm caused by the virus and alleviating inflammation.^[36]^ A single intravenous injection of bone marrow-derived EVs has been demonstrated to profoundly reverse hypoxia, immune reconstruction, and cytokine storm in severe coronavirus disease 2019 (COVID-19) cases without any treatment-related adverse reactions.^[37]^ Additionally, EVs are involved in the regulation of TNF-α levels. In preclinical models of ARDS, EVs can transfer to macrophages and inhibit TNF-α production in alveolar macrophages. Furthermore, mitochondrial transfer from mesenchymal SCs has been shown to significantly reduce inflammatory cytokines, improve mitochondrial function in alveolar cells, and alleviate inflammation induced by asthma.^[9]^

### 3.4. Targeted tumor diagnosis and therapy

EVs liquid biopsy is a method of inferring the prognosis of cancer patients by analyzing the components of EVs. The molecular markers present in EVs can be used as indicators for early cancer detection, cancer staging, and prognostic evaluation.^[38]^ It is a highly promising diagnostic and therapeutic method. For example, studies have demonstrated that circRNA exosomal membrane protein 1 of EVs in patient serum samples can serve as a novel biomarker for non-small cell lung cancer, and it promotes non-small cell lung cancer proliferation by regulating the miR-524-5p-METTL3/SOX2 axis. A scholar at New York University provided a comprehensive summary and elaboration on the relevant research on EVs in tumor progression over the past decade,^[39]^ emphasizing the significant role played by EVs in the formation of pre-metastatic niches in tumor metastasis.^[40]^ Initial research has demonstrated that EVs derived from cancer cells impede the maturation and migration of dendritic cells via the PD-L1-dependent pathway, thereby promoting T-cell exhaustion and reducing their antitumor efficacy.^[41]^ Emerging evidence highlights MSC-EVs as pivotal mediators of intercellular communication that orchestrate tumor progression through multifaceted mechanisms. Their pathological roles have been substantiated across multiple malignancies, including lung cancer, breast cancer,^[42]^ nasopharyngeal carcinoma,^[43]^ and osteosarcoma.^[44]^ MSC-EVs have been shown to not only promote tumor angiogenesis, but also play an important role in cancer development and metastasis by regulating immune cell function in the tumor microenvironment and influencing tumor cell dormancy and activation.^[45,46]^ These innate nanovesicles exhibit tissue targeting specificity and exceptional biocompatibility. Notably, their surface membrane proteins can be meticulously engineered through modular biotechnological methodologies, including protein engineering,^[47]^ antibody-drug conjugation,^[48]^ genome editing,^[49]^ and lipid assembly.^[50]^ These findings position MSC-EVs as programmable platforms for the development of next-generation targeted anticancer therapeutics. Recent research has also corroborated the findings of Chen et al,^[51]^ who demonstrated that inhibiting the release of PD-L1 EVs by knocking out the ORAI1 gene enhances their antitumor effect. Furthermore, microRNA-25-3p, microRNA-155-5p, microRNA-215-5p, and microRNA-375 present in EVs derived from CD4 T cells have been demonstrated to influence CD8 T cell proliferation and activity, thereby enhancing their antitumor effect.^[52]^ This may provide a foundation for new research directions, whereby scientists could utilize engineered T cells to block relevant cancer immune checkpoints, alter the function and phenotype of tumor cells, and inhibit cancer onset.

### 3.5. Investigational applications of extracellular vesicles in respiratory clinical trials

EVs demonstrate multimodal regulatory capacity across respiratory pathologies, showing therapeutic efficacy in diverse pulmonary diseases.^[5]^ Notably, in COVID-19, multicenter clinical trials reveal significantly improved clinical outcomes with EV-based interventions compared to the control group, regardless of nebulized inhalation^[53]^ or intravenous injection^[54]^ administration routes. This therapeutic superiority positions EV biologics as promising next-generation interventions for severe viral pneumonia. Furthermore, a comprehensive analysis of safety data from recent multicenter, double-blind, randomized controlled trials has demonstrated that no significant adverse events related to the intervention were reported in the exosome-treated group, thereby providing substantial safety support for the clinical translation of MSC-EVs therapy.^[54,55]^ Recent clinical findings suggest that exosome-engineered multi-allogeneic protein systems (Exo-d-MAPPS) have the potential to serve as innovative biologics for the management of COPD. These biologics have been shown to orchestrate immunomodulation by substantially suppressing inflammatory cytokine secretion from pulmonary-infiltrating immune subsets, including alveolar macrophages, neutrophilic granulocytes, and natural killer cells. This targeted immunoregulatory process consequently ameliorates both pathological lung remodeling and clinical symptoms in COPD patients.^[56]^ In the domain of oncology, EVs, in conjunction with circulating tumor DNA (ctDNA), serve as a significant molecular marker for liquid biopsy. Research has demonstrated that ctDNA mutations are discernible in the peripheral blood of approximately 37% of lung cancer patients, and their mutation profiles exhibit a high degree of consistency with tissue biopsy results. As an emerging liquid biopsy marker, the combined detection of EVs is expected to provide a more comprehensive molecular characterization of patients with metastatic non-small cell lung cancer57, thereby optimizing prognostic assessment and guiding individualized treatment decisions.^[57]^

## 4. Limitations and challenges

EVs have emerged as pivotal mediators of intercellular communication, exhibiting considerable promise in the domains of disease diagnosis, therapy, and drug delivery.^[58]^ The successful application of EVs signifies a state-of-the-art technology for cell-free therapies. Nevertheless, the field remains in a phase of rapid development, with the technology still in its nascent stages. A multitude of technological impediments and challenges persist in the process of translating it from fundamental research to clinical application. The ensuing systematic analysis encompasses the 4 major dimensions of EVs isolation, large-scale production, stability and storage, and heterogeneity: the limitations of isolation techniques: at present, the most common methods of extracellular vesicle extraction include differential ultracentrifugation, density gradient ultracentrifugation, immunomagnetic bead capture, tangential flow filtration, size exclusion chromatography, and aptamer-based microfluidic biosensors. A comparison of the advantages and disadvantages of these mainstream separation techniques is provided in Table 1. A common challenge faced by these methods is the difficulty in achieving both purity and yield. Of particular concern is the absence of an international standard for quality control, which hinders the comparability of data from disparate studies. bottlenecks and risks of large-scale production: the large-scale production of EVs faces significant challenges, primarily due to limitations in cell sources and expansion capabilities. For instance, mesenchymal SCs, a prevalent source of EVs, exhibit constrained proliferative potential, with finite passage numbers that impede the attainment of sustainable production levels. Furthermore, a critical consideration in the large-scale manufacturing of EVs is cost-effectiveness, which necessitates a balanced approach between yield, purity, and economic viability. Addressing these challenges necessitates further research to optimize production processes, enhance scalability, and reduce costs while maintaining high EVs yield and quality. Stability and storage: it is important to note that MSC-EVs currently exhibit limited physicochemical stability, making them susceptible to aggregation and membrane rupture during freeze-thaw cycles. Furthermore, in vivo applications are confronted with 2 substantial challenges: enzymatic degradation and rapid immune clearance. The aforementioned factors collectively compromise the structural integrity and functional efficacy of MSC-EVs, posing significant challenges for their therapeutic use and long-term storage. Heterogeneity: the issue of heterogeneity represents a significant impediment, arising from variations in isolation methodologies and the biological characteristics of the extracellular vesicle sources. For instance, in the context of lung cancer therapy, the presence of heterogeneous EV populations can compromise the efficacy of drug delivery, resulting in suboptimal target tissue enrichment and increased off-target uptake by healthy tissues. This, in turn, can lead to a reduction in therapeutic efficacy and the potential for adverse effects. Furthermore, while MSC-derived EVs show promise for immunomodulation, their long-term immunological impacts remain poorly understood. Currently, there is a critical lack of clinical safety data regarding prolonged EV exposure, necessitating rigorous large-scale trials and longitudinal studies to evaluate potential risks.

## 5. Conclusions

The successful application of EVs as a noncellular therapy represents a cutting-edge technology. They play a role in tissue repair, immune regulation, targeted tumor diagnosis and therapy activity. As a novel formulation strategy for EVs, they facilitate the delivery of soluble proteins via noninvasive administration routes. Nevertheless, the research is still in its infancy, and the technology is not yet fully developed. Numerous challenges still need to be addressed. These include determining the optimal timing window, administration route, and frequency for delivering EVs, identifying the most effective method for large-scale production, developing a reliable process for reconstructing frozen cells for clinical use and conducting rigorous cell potency testing. These issues require further investigation and the formulation of solutions by the research community.

## Author contributions

**Conceptualization:** Tingting Zu.

**Data curation, Investigation:** Tingting Zu, Meng Gao, Baohe Liu, Xuejing Zhang.

**Writing – original draft:** Tingting Zu, Baohe Liu.

**Writing – review & editing:** Fuling Wu.
